# Sunscreen-Based Photocages for Topical Drugs: A Photophysical and Photochemical Study of A Diclofenac-Avobenzone Dyad

**DOI:** 10.3390/molecules23030673

**Published:** 2018-03-16

**Authors:** Isabel Aparici-Espert, Miguel A. Miranda, Virginie Lhiaubet-Vallet

**Affiliations:** Instituto Universitario Mixto de Tecnología Química, Universitat Politècnica de València, Consejo Superior de Investigaciones Científicas, Avda de los Naranjos, s/n, 46022 Valencia, Spain; iapariciespert@gmail.com

**Keywords:** photochemistry, photolabile protecting group, photoprotection, phototoxicity

## Abstract

Photosensitization by drugs is a problem of increasing importance in modern life. This phenomenon occurs when a chemical substance in the skin is exposed to sunlight. Photosensitizing drugs are reported to cause severe skin dermatitis, and indeed, it is generally advised to avoid sunbathing and to apply sunscreen. In this context, the nonsteroidal anti-inflammatory drug (NSAID) diclofenac is a photosensitive drug, especially when administered in topical form. In this work, efforts have been made to design and study an innovative pro-drug/pro-filter system containing diclofenac and the UVA filter avobenzone in order to develop a safer use of this topical drug. The design is based on the presence of a well-established photoremovable phenacyl group in the avobenzone structure. Steady-state photolysis of the dyad in hydrogen-donor solvents, monitored by UV-Vis spectrophotometry and HPLC, confirms the simultaneous photorelease of diclofenac and avobenzone. Laser flash photolysis and phosphorescence emission experiments allow us to gain insight into the photoactive triplet excited-state properties of the dyad. Finally, it is shown that avobenzone provides partial photoprotection to diclofenac from photocyclization to carbazole derivatives.

## 1. Introduction

The harmful effect of sunlight is becoming a public health concern due to the increased incidence of skin cancer [1,2,3]. This has been associated with sun exposure in connection with sun-seeking behavior, tanning fashion, and outdoor activities. Fortunately, the main UV-component of sunlight—UVA, which includes wavelengths ranging from 320 to 400 nm—is hardly absorbed by biomolecules. However, UVA is an important factor in the process of chemical photosensitization, during which light does not interact directly with biological components, but is absorbed by endogenous or exogenous compounds that can act as photodamaging agents [4,5,6,7].

Topical drugs are of special relevancy as they are applied to the skin’s surface, and thus, they are directly exposed to sunlight irradiation without suffering the filtering effect provided by the dermis and epidermis. In this context, nonsteroidal anti-inflammatory drugs (NSAIDs) contained in topical pain relievers are of widespread use for the treatment of musculoskeletal disorders. Topical formulations containing diclofenac (DCF) as the pharmacologically active agent are among the most commonly utilized because they are available in many countries without the need for a medical prescription. The topical form generally exhibits a better safety profile than the systemic form, as it reduces the diffusion of the drug in the organism, thus avoiding the appearance of the most common side effects. However, direct application of the drug is not always harmless and is at the origin of skin photosensitivity. In this context, several cases of photoallergic contact dermatitis have been reported for DCF as a topical pain reliever [8,9,10,11,12,13], but also when it is the active component in the treatment of actinic keratosis [14].

**Scheme 1:**
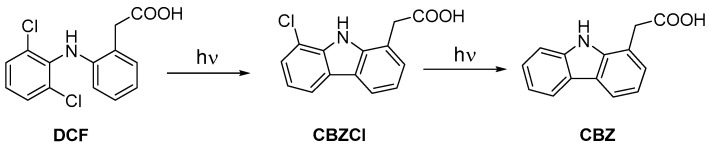
Structures of diclofenac (DCF) and its main carbazole-derived photoproducts.

Photochemical studies have revealed that diclofenac is not stable under irradiation, and it undergoes a photocyclization process that gives rise to a primary photoproduct that is a chlorocarbazole derivative (CBZCl), which in turn photodechlorinates to yield the corresponding carbazole (CBZ), as illustrated in Scheme 1 [15,16,17]. In vitro experiments have shown that CBZCl is the main contributor to lipid peroxidation through a radical-mediated mechanism, while DCF participates to a lesser extent [16]. This difference may be exaggerated by the fact that carbazoles exhibit higher UVA absorption than diphenylamine derivatives, and thus are more prone to be excited by sunlight radiation that causes them to reach their photoreactive states [15].

Indeed, the medical leaflet for diclofenac gel contains explicit warnings regarding its photoreactivity, which advise the patient to minimize or avoid natural or artificial sun exposure of treated areas. Recent studies have shown that the photostability of DCF can be enhanced by improving its topical formulation and by including solar filters [18,19]. In this context, we have designed a new pro-drug/pro-filter compound that contains another photosensitizing NSAID, namely ketoprofen, and its complementary UVA filter avobenzone [20]. This dyad takes advantage of the diketo tautomer of avobenzone (AB, 4-*tert*-butyl-4′-methoxydibenzoylmethane) as a phenacyl-like photolabile protecting group that is able to photorelease carboxylic acid derivatives [21,22,23,24]. Avobenzone is widely used as a UVA filter in sunscreen and cosmetic preparations. In solution, as with most of dibenzoylmethane derivatives, the tautomeric equilibrium is displaced towards the keto-enol form, which is responsible for its high UVA absorption. Thus, the concept is based on the fact that the dyad is cleaved through irradiation, photoreleasing the phototoxic drug and its protective UVA shield. 

**Scheme 2:**
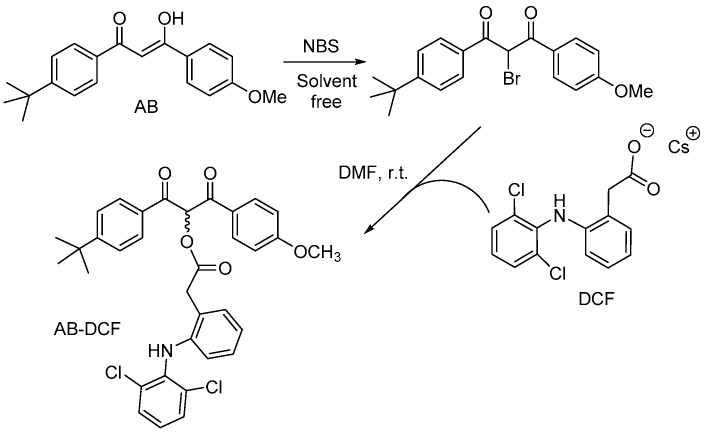
Synthetic scheme of the diclofenac/avobenzone photoactivatable dyad (AB-DCF).

In this work, a similar approach has been envisaged for DCF. This time the diketo form present in the photoactivatable dyad should afford protection to DCF, while the keto-enol tautomer overlaps the UVA-absorption region of the carbazole-like photoproducts. Thus, the designed dyad AB-DCF, which contains the diclofenac anchored to the α-position of the AB diketo groups (Scheme 2), should release the active drug and its associated UVA-filter AB after being exposed to simulated sunlight (SSL) irradiation. This possibility has been checked by examining the progress of the photorelease by means of UV-Vis absorption, fluorescence emission, and HPLC. In addition, phosphorescence emission and laser flash photolysis experiments have also been performed to gain more insight into the photoactive triplet excited state of the dyad.

## 2. Results

### 2.1. Synthesis

The synthesis of the dyad AB-DCF was performed in two steps (Scheme 2) [20]. Briefly, the α-bromo avobenzone derivative was first obtained under solvent-free conditions using *N*-bromosuccinimide as the brominating agent, following the described procedure [25]. Then, the resulting product was reacted with diclofenac cesium salt to afford the desired dyad AB-DCF as an enantiomeric mixture. The NMR spectra (^1^H, ^13^C, COSY, and HSQC) showed that the avobenzone moiety was present under its diketo form. Indeed, the characteristic proton signal at 6.75 ppm of the “chelated” enol form was shifted to 6.94 ppm (see Supplementary Materials). This assignment was confirmed by 2D NMR spectroscopy showing that this 6.94 ppm proton correlated with the α-dicarbonyl carbon at 81.2 ppm (see Supplementary Materials).

**Figure 1:**
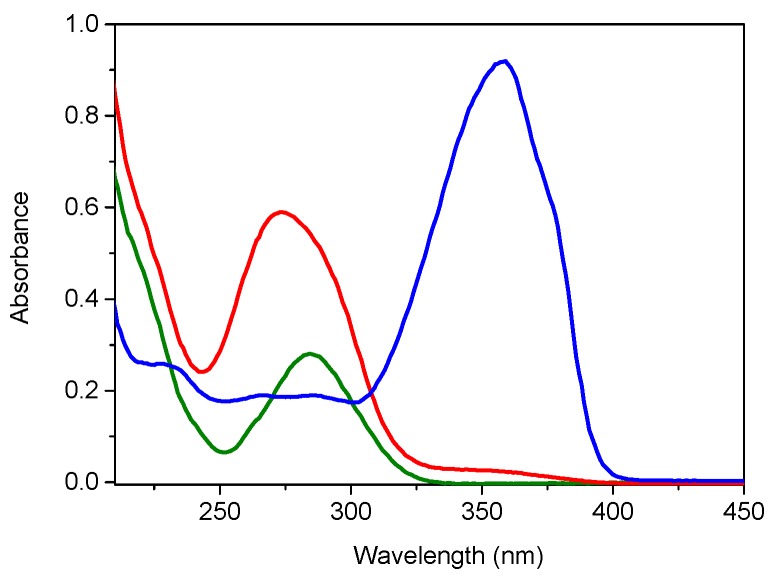
UV-Vis absorption spectra of avobenzone (AB, blue), diclofenac (DCF, green), and the dyad AB-DCF (red) in ethanol at ca. 1.5 × 10^−5^ M.

Accordingly, the strong absorption of the avobenzone in its ‘‘chelated’’ enol form, with maximum absorption at ca. 340–350 nm, was not detected in the UV-Vis absorption spectrum of AB-DCF (Figure 1). Instead, a peak centered at 275 nm, which accounted for the absorption of the diclofenac moiety together with that of the diketo tautomer of the avobenzone part, was observed together with a shoulder that almost reached 390 nm. The presence of this UVA-absorbing portion was of paramount importance in order to achieve a sunlight-triggered photorelease of the drug and the filter.

### 2.2. Photorelease Experiments

#### 2.2.1. UV-Vis Absorption

**Figure 2:**
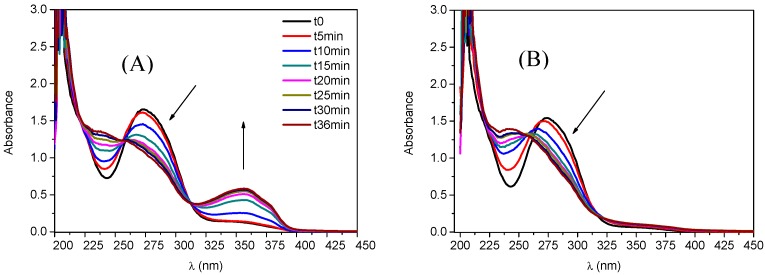
Steady-state photolysis of the AB-DCF ethanolic solution (4.3 × 10^−5^ M) by means of simulated sunlight (SSL) under N_2_ (**A**) and air (**B**) atmospheres followed by UV-Vis spectrophotometry.

Steady-state photolysis of AB-DCF was performed under SSL conditions. Ethanolic solutions of the dyad (4.3 × 10^−5^ M) were irradiated under aerobic or anaerobic conditions. Owing to the characteristic UV absorbance of the “chelated” avobenzone, its photorelease was easily followed by UV-Vis absorption. Very different spectral changes were registered for air- or nitrogen-equilibrated solutions (Figure 2). Indeed, under deaerated conditions, the maximum absorption at 275 nm was shifted and decreased during irradiation, while the 355 nm band increased. By contrast, under aerated conditions, the decrease of the maximum absorption did not result in the formation of a new UVA band. These results led us to look for a reaction medium more similar to the in vivo application conditions.

**Figure 3:**
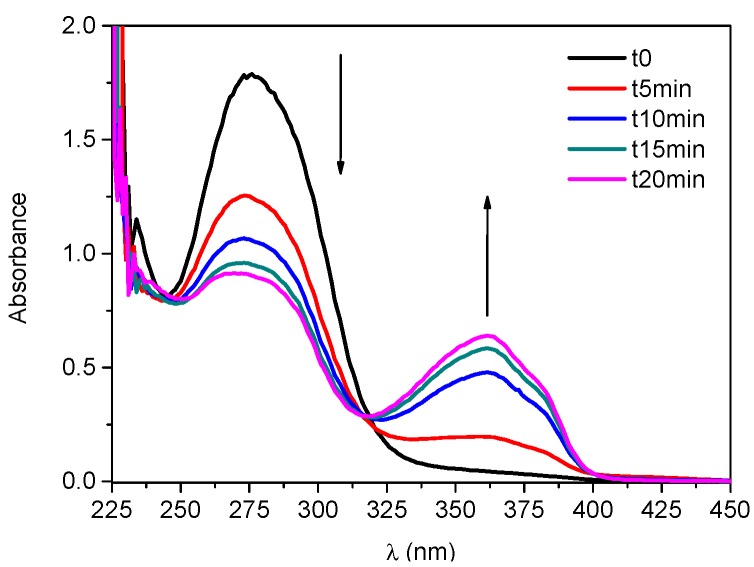
UV-Vis absorption spectra of an aerated diethylene glycol (dEtGly) solution of AB-DCF (6.6 × 10^−5^ M) irradiated using SSL.

**Scheme 3:**
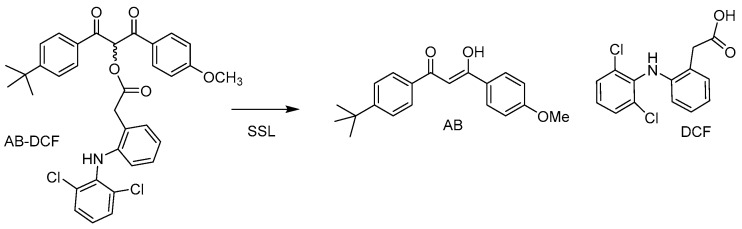
Photorelease of AB and DCF from the dyad (AB-DCF).

Therefore, an alternative approach was envisaged to improve air-equilibrated experiments. It was based on using a hydrogen-donor solvent with properties such as low O_2_ solubility, high viscosity, and thus, a low diffusion-controlled rate constant, which altogether was thought to favor the photorelease of diclofenac and avobenzone (Scheme 3). Diethylene glycol (dEtGly) appeared as a matrix of interest as it fulfilled the preceding points; moreover, it is an ingredient (co-solvent) in topical drug or healthcare product formulations. Irradiation of an aerated solution of AB-DCF (6.6 × 10^−5^ M) in dEtGly confirmed that the solvent choice was appropriate, as observed from the absorption spectra shown in Figure 3, which exhibited the characteristic growth of the avobenzone band at 355 nm.

#### 2.2.2. HPLC Analysis

**Figure 4:**
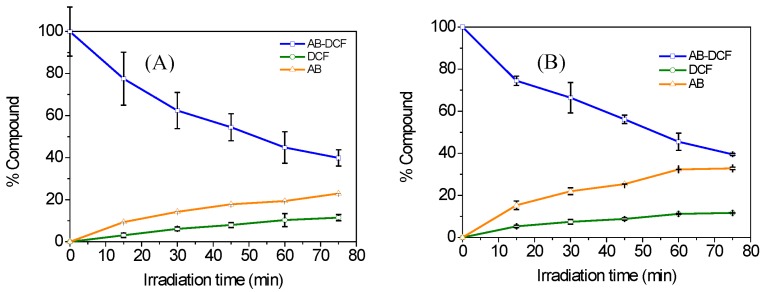
Time course of SSL irradiated samples of (**A**) AB-DCF in EtOH/N_2_, (**B**) AB-DCF in dEtGly/air.

The experiments using UV-Vis spectrophotometry gave us a first insight into the viability of our system. However, the superposition of the absorption spectrum of DCF with that of the initial dyad (Figure 1) did not allow a clean quantitation of the drug release. Thus, HPLC experiments were carried out to overcome this limitation. The quantitation of the different compounds was achieved by using authentic samples of AB-DCF, AB, and DCF for calibration. Upon SSL irradiation of AB-DCF in deaerated EtOH, almost 60% of the dyad was consumed after 60 min, giving rise to DCF and AB formations (Figure 4A). The same tendency, with a somewhat higher release of AB, was observed for an aerated solution of DCF in dEtGly as shown in Figure 4B. 

#### 2.2.3. Fluorescence Analysis

**Figure 5:**
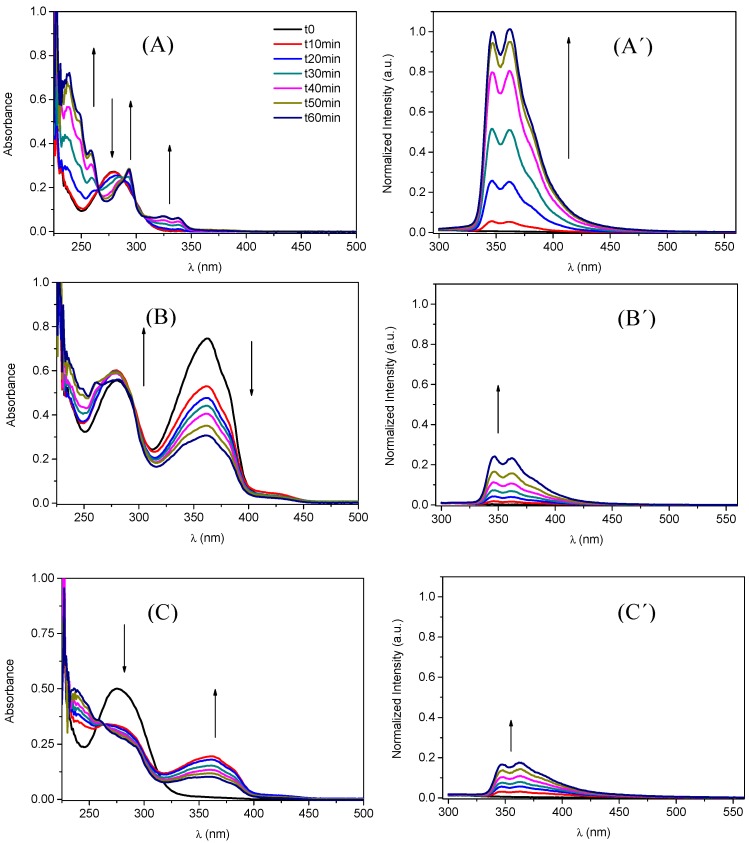
UV-Vis spectra (left (**A**–**C**)) and fluorescence emission spectra (right, (**A’**–**C’**)) of DCF alone (**A**,**A’**), the equimolar DCF+AB mixture (**B**,**B’**), and the AB-DCF dyad (**C**,**C’**) obtained during the SSL irradiation of aerated diethylene glycol solutions (1.7 × 10^−5^ M).

**Figure 6:**
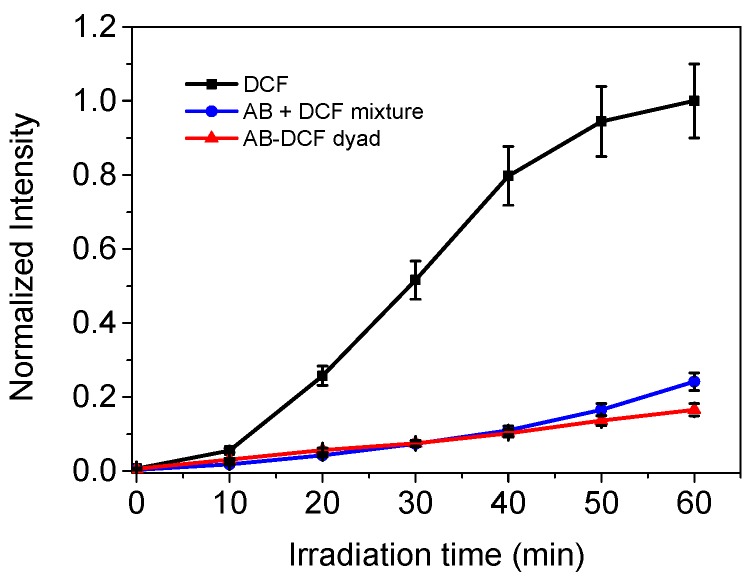
Time-course formation of carbazole-derived photoproducts followed by fluorescence, registering the emission intensity at 347 nm (λ_exc_ = 290 nm) for aerated solutions of DCF alone (black), the equimolar DCF+AB mixture (blue), and the AB-DCF dyad (red) in diethylene glycol.

As mentioned above, the phototoxic properties of diclofenac are due to the formation of a chlorocarbazole photoproduct CBZCl, and its subsequent photodehalogenation to CBZ. Formation of these photoproducts can be easily followed by fluorescence emission as, in contrast with the parent drug, they exhibit a fluorescence emission with λ_em_ at ca. 350 and 360 nm [15]. Thus, SSL irradiations were carried out in aerated dEtGly solutions of DCF alone, DCF in the presence of AB (1:1, DCF+AB), and AB-DCF (Figure 5). Under irradiation, the maximum absorption of the drug at 270 nm decreased concomitantly with the increase of red-shifted bands with λ_max_ at ca. 294, 325, and 336 nm (Figure 5A), which is consistent with the formation of the carbazole-derived photoproducts [15]. Concerning the absorption measurements of the equimolar mixture DCF+AB or the dyad, the UVA band of the keto-enol form of AB overlapped with those of the photoproducts (Figure 5B,C), preventing them from following their kinetics of formation. This difficulty was anticipated, as AB was expected to provide photoprotection to the photoproducts. Fortunately, the non-fluorescent AB did not interfere in the emission measurements, allowing us to monitor the photoproducts formation. As shown in Figure 5A’–C’, the increased emission intensity due to the carbazole derivatives was markedly lower for the equimolar DCF+AB mixture, as well as for the dyad AB-DCF. This is clearly illustrated in Figure 6 by plotting of the fluorescence intensity (at λ_em_ ca. 367 nm) as a function of the irradiation time.

In summary, fluorescence monitoring of the irradiations reveals that, both in the equimolar DCF+AB mixture and in the AB-DCF dyad, partial protection was provided by the preexisting or nascent keto-enol form of the filter, although formation of the carbazole-derived photoproduct was not completely prevented. The advantage of using the dyad instead of the mixture is related to the controlled release of the active principle.

### 2.3. Time-Resolved Experiments

**Figure 7:**
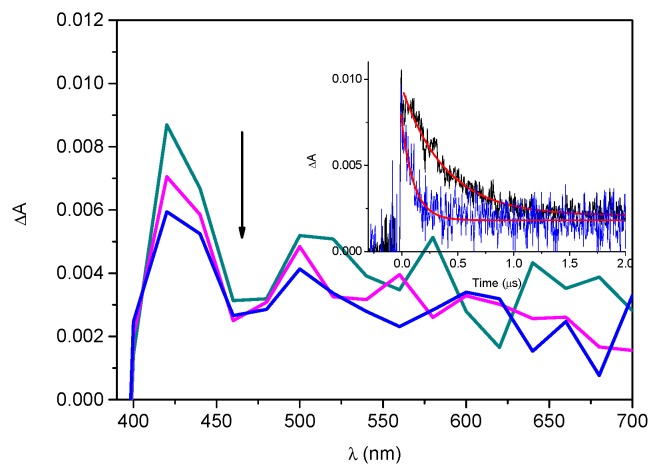
Transient absorption spectra of the AB-DCF dyad (6.4 × 10^−4^ M) in ethanol under N_2_, from 0.1 to 0.27 µs after the 355 nm laser pulse. Inset: decays monitored at 420 nm under N_2_ (black) or air (blue) and their corresponding fittings (red).

Laser flash photolysis was carried out to obtain information on the transient species generated during excitation at room temperature. Thus, for N_2_-bubbled solutions of AB-DCF, only one species was observed immediately after the 355 nm laser pulse (Figure 7). This species, with a lifetime (τ) of ca. 0.4 μs, was quenched by oxygen; under air-equilibrated conditions, the determined τ was of ca. 0.1 μs (Figure 7, inset). This transient was similar to that previously described for α-methylated AB (AB–Me) [26] and for the dyad containing AB and ketoprofen [20] and was thus assigned to the triplet excited state of AB-DCF. Moreover, a comparison of the lifetime in the case of the photolabile dyad (τ of ca. 0.4 μs) and that of the α-methyl derivative of AB (τ = 0.8 μs) suggests that the triplet excited state was the involved species in the photorelease of AB and DCF.

**Figure 8:**
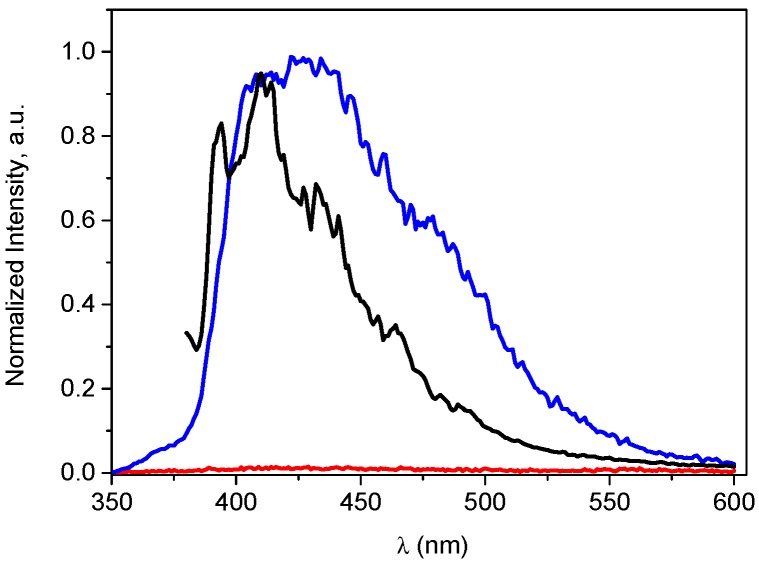
Phosphorescence emission spectra of DCF (red), the AB-DCF dyad (blue), and AB–Me (black) in ethanol at 77 K at the excitation wavelength λ_exc_ of ca. 284 nm.

In order to obtain additional information about the triplet excited state of AB-DCF, its phosphorescence emission spectrum was recorded at 77 K, and a broad band was observed with a maximum at ca. 430 nm (Figure 8). Interestingly, DCF did not show any phosphorescence emission under the same conditions, so this moiety did not contribute to the obtained spectrum of AB-DCF. The emission of AB-DCF was somewhat red-shifted and less structured with respect to that of the previously described phosphorescence of AB–Me, used as reference to illustrate AB in its diketo form [26]. However, this was consistent with a change in the α-substitution—alkyl chain versus ester moiety.

## 3. Materials and Methods

### 3.1. Reagents and Solvents

Avobenzone, *N*-bromosuccinimide, sodium diclofenac salt, anhydrous dimethylformamide, hexane, and ethyl acetate were purchased from AK Scientific (Union City, CA, USA) and Sigma-Aldrich (St. Louis, MO, USA) and used as received. The ethanol of HPLC grade, used for the steady-state photolysis and laser flash photolysis experiments, was from Scharlau (Barcelona, Spain), whereas the diethylene glycol was purchased from Panreac (Barcelona, Spain).

### 3.2. Characterization

The ^1^H and ^13^C NMR spectra were measured by a 300 MHz instrument (Bruker, Rivas-Vaciamadrid, Spain), and CDCl_3_ was used as the solvent for all the spectra. The solvent signal was taken as the reference using a chemical shift (δ) of ca. 7.26 ppm and 77.16 ppm for ^1^H and ^13^C NMR, respectively. Coupling constants (*J*) were given in Hz. The exact mass values were determined by using a Waters ACQUITY™ XevoQToF spectrometer (Waters Corp., Cerdanyola del Vallès, Spain) connected to the UPLC system via an electrospray ionization (ESI) interface. The ESI source was operated in positive ionization mode with the capillary voltage at 3.0 kV (Waters Corp., Cerdanyola del Vallès, Spain). The temperature of the source and desolvation was set at 120 °C and 500 °C, respectively. The cone and desolvation gas flows were 10 L h^−1^ and 450 L h^−1^, respectively. All data collected in centroid mode were acquired using Masslynx™ software (Waters Corp., Cerdanyola del Vallès, Spain). Leucine-enkephalin was used as the lock mass, generating an [M + H]^+^ ion (*m*/*z* 556.2771) at a concentration of 250 pg/mL and a flow rate of 20 μL/min to ensure accuracy during the mass analysis.

### 3.3. Synthesis

*2-bromo-1-(4-(tert-butyl)phenyl)-3-(4-methoxyphenyl)propane-1,3-dione (AB-Br)*. Two solids, avobenzone (AB, 1 g, 3.2 mmol) and *N*-bromosuccinimide (0.57 g, 3.2 mmol) were crushed under solvent-free conditions in a mortar for 10 min. The mixture was left standing for 2 hours and then mashed each 15 min for 2 h. Then, water was added, and the solution was filtered under a vacuum. The solid was dried and purified by flash chromatography, using hexane:ethyl acetate as an eluent (6:1, *v*:*v*). Colorless crystals of AB-Br were obtained (0.94 g) in a 76% yield. ^1^H NMR (300 MHz, D_2_O) δ: 7.99 (d, 2H, *J* = 9 Hz), 7.93 (d, 2H, *J* = 9 Hz), 7.46 (d, *J* = 6 Hz, 2H), 6.92 (d, *J* = 6 Hz, 2H), 6.47 (s, 1H), 3.86 (s, 3H), 1.32 ppm (s, 9H). ^13^C NMR (75 MHz, D_2_O) *δ*: 188.7 (CO), 187.7 (CO), 164.4 (C), 158.2 (C), 131.9 (CH), 131.3 (C), 129.3 (CH), 126.8 (C), 126.1 (CH), 114.3 (CH), 55.7 (CH_3_), 53.3 (CH), 35.3 (C), 31.0 ppm (CH_3_). HMRS (ESI-TOF): *m*/*z* [M + H]^+^ calculated for C_20_H_22_O_3_Br: 389.0752 found: 389.0753.

*1-(4-(tert-butyl)phenyl)-3-(4-methoxyphenyl)-1,3-dioxopropan-2-yl 2-(2-((2,6-dichlorophenyl)amino) phenyl)acetate (AB-DCF)*. Sodium diclofenac salt (0.18 g, 0.567 mmol) was added to a solution of AB-Br (0.2 g, 0.515 mmol) in DMF under an inert atmosphere, and it was stirred at room temperature for 22 h. The DMF was evaporated under vacuum, and the crude product was purified by column flash chromatography using hexane: ethyl acetate (9:1, *v*:*v*) as an eluent. The dyad AB-DCF was obtained as a yellowish solid—an enantiomeric mixture in 50% yield. ^1^H NMR (300 MHz, CDCl_3_) δ: 8.01 (d, *J* = 9 Hz, 2H), 7.96 (d, *J* = 6 Hz, 2H), 7.42 (d, *J* = 9 Hz, 2H), 7.31 (d, *J* = 9 Hz, 2H), 7.24 (dd, *J* = 6 Hz, *J* = 1 Hz, 1H), 7.13 (m, 1H), 6.98 (d, *J* = 9 Hz, 2H), 6.94 (s, 1H), 6.86 (d, *J* = 9 Hz, 2H), 6.54 (dd, *J* = 9Hz, *J* = 1 Hz, 1H), 6.46 (bs, 1H), 4.03 (s, 2H), 3.83 (s, 3H), 1.30 (s, 9H). ^13^C NMR (75 MHz, CDCl_3_) δ: 190.5 (CO), 189.2 (CO), 170.7 (COO), 164.4 (C), 158.2 (C), 143.0 (C), 138.2 (C), 132.2 (2CH), 131.8 (C), 131.2 (CH), 129.7 (2 C), 129.6 (2 CH), 128.9 (2 CH), 128.3 (CH), 127.4 (C), 125.8 (2 CH), 124.4 (C), 124.1 (CH), 122,6 (CH), 119.1 (CH), 114.1 (2 CH), 81.2 (CH, diketonic), 55.6 (CH_3_, –OMe), 38.2 (CH_2_), 35.3 (C), 31.1 (3 CH_3_). HMRS (ESI-TOF): *m*/*z* [M + H]+ calculated for C_34_H_32_Cl_2_NO_5_: 604.1658 found: 604.1660.

### 3.4. Photophysical/Photochemical Instrumentation

#### 3.4.1. UV-Vis Absorption

The spectra were registered on a Cary 50 spectrophotometer (Varian, Palo Alto, CA, USA) using a quartz cuvette of 3 mL and a 1 cm optical path. 

#### 3.4.2. Fluorescence Emission

The steady-state fluorescence experiments were carried out by means of a Photon Technology International (PTI) LPS-220B spectrofluorometer (PTI, Birmingham, NJ, USA), equipped with an Xe lamp (75 W) and a monochromator covering the region of 200–700 nm. The excitation λ_exc_ was 290 nm and an emission was registered from 300 to 560 nm. Irradiations were carried out in diethylene glycol at ca. 1.7 × 10^−5^ M in SSL, and the reaction was monitored in the spectrofluorometer.

#### 3.4.3. Phosphorescence Emission

The phosphorescence spectra were registered with a Photon Technology International (PTI, TimeMaster TM/2003, Birmingham, NJ, USA) equipped with a pulsed Xenon lamp. The equipment worked in time-resolved mode with a delay time of 500 µs. Compounds were dissolved in ethanol solutions with an absorbance of 0.8 at the excitation wavelength of 284 nm (value determined for a 1 cm optical path). Then, the solution was transferred to a quartz tube of 5 mm diameter, and measurements were performed at 77 K.

#### 3.4.4. Laser Flash Photolysis (LFP)

The experiments were run with a pulsed Nd:YAG (L52137 V LOTIS TII, Minsk, Belarus) laser system instrument setting 355 nm as the excitation wavelength. The pulse duration was of ca. 10 ns, and the energy was adjusted at 26 mJ pulse^−1^. The apparatus consisted of the pulsed laser, the Xe lamp, a 77250 Oriel monochromator, and a photomultiplier. The output signal from a Tektronix (Les Ulis, France) oscilloscope was transferred to a personal computer. The transient spectra were recorded at room temperature, employing quartz cells of 1 cm optical path length. Experiments were performed for ethanol solutions of AB-DCF (6.4 × 10^−4^ M) under air and N_2_ atmospheres. 

#### 3.4.5. Steady-State Photolysis

The simulated sunlight irradiation was performed by a Thermo Oriel Newport (A91192A) (Newport, CA, USA) solar simulator equipped with a 1000 W Xe arc. Its output was adequately filtered to produce a spectrum approximating natural sunlight (1.5 G air mass filter). The spectral output was measured as ca. 100 mW/cm^2^. Experiments were carried out in deaerated or aerated ethanol solutions of AB-DCF at ca. 4.3 × 10^−5^ M and in aerated diethylenglycol at ca. 6.6 × 10^−5^ M. Finally, HPLC (Varian, Palo Alto, CA, USA) analysis was run using SSL irradiation of AB-DCF at 1.0 × 10^−3^ M or 9.3 × 10^−4^ M in deaerated ethanol or aerated diethylene glycol, respectively.

#### 3.4.6. HPLC Analysis

The irradiation mixtures were analyzed by reversed-phase HPLC using a Varian ProStar instrument equipped with a diode array detector covering a detection range from 200 to 400 nm. A Mediterranea Sea C18 column (25 mm × 0.46 mm, 5 µm) (Teknokroma, Sant Cugat, Spain) was employed with an injection volume of 10 μL (removed from the cuvette at different irradiation times). The mobile phase was an isocratic mixture of water at pH 3 (20%) and acetonitrile (80%) at a flow rate of 0.7 mL min^−1^. Different detection wavelengths were used to detect AB-DCF (λ_detection_ = 274 nm), DCF (λ_detection_ = 282 nm), or AB (λ_detection_ = 357 nm).

## 4. Conclusions

In this work, the pro-drug/pro-filter concept has been checked for a covalently linked dyad constructed from the NSAID diclofenac (DCF) and the widely used solar filter avobenzone (AB). Exposure of the dyad (AB-DCF) to solar simulated light in diethylene glycol under aerobic conditions led to the simultaneous release of the two components DCF and AB, demonstrating the effective photorelease of the drug from the photocage. In addition, the nascent AB provided partial photoprotection to DCF, which underwent photocyclization to carbazole derivatives much slower than it did in the absence of the solar filter. Our current work is directed toward optimizing the photorelease yield, improving photoprotection, and testing permeation capability.

## Supplementary Materials

The following are available online. ^1^H NMR and ^13^C NMR spectra of AB-DCF, DEPT and HSQC spectra of AB-DCF, and HPLC chromatograms of AB-DCF irradiated in deaerated ethanol.
