# Supplementary material for: Sunscreen-Based Photocages for Topical Drugs: A Photophysical and Photochemical Study of A Diclofenac-Avobenzone Dyad
Source: Molecules. 2018 Mar 16;23(3):673. doi: 10.3390/molecules23030673 (PMC6017856; doi:10.3390/molecules23030673)
Supplement: Supplementary file 1 [file molecules-23-00673-s001.pdf]

Supplementary Materials

Sunscreen-based Photocages for Topical Drugs: A Photophysical and Photochemical Study of A Diclofenac-Avobenzone Dyad

*Isabel Aparici-Espert, Miguel A. Miranda * and Virginie Lhiaubet-Vallet **

Index:

S2 ^{1}H NMR and ^{13}C NMR spectra of AB-DCF

S3 DEPT and HSQC spectra of AB-DCF

S4 HPLC chromatograms of AB-DCF irradiated in deaerated ethanol

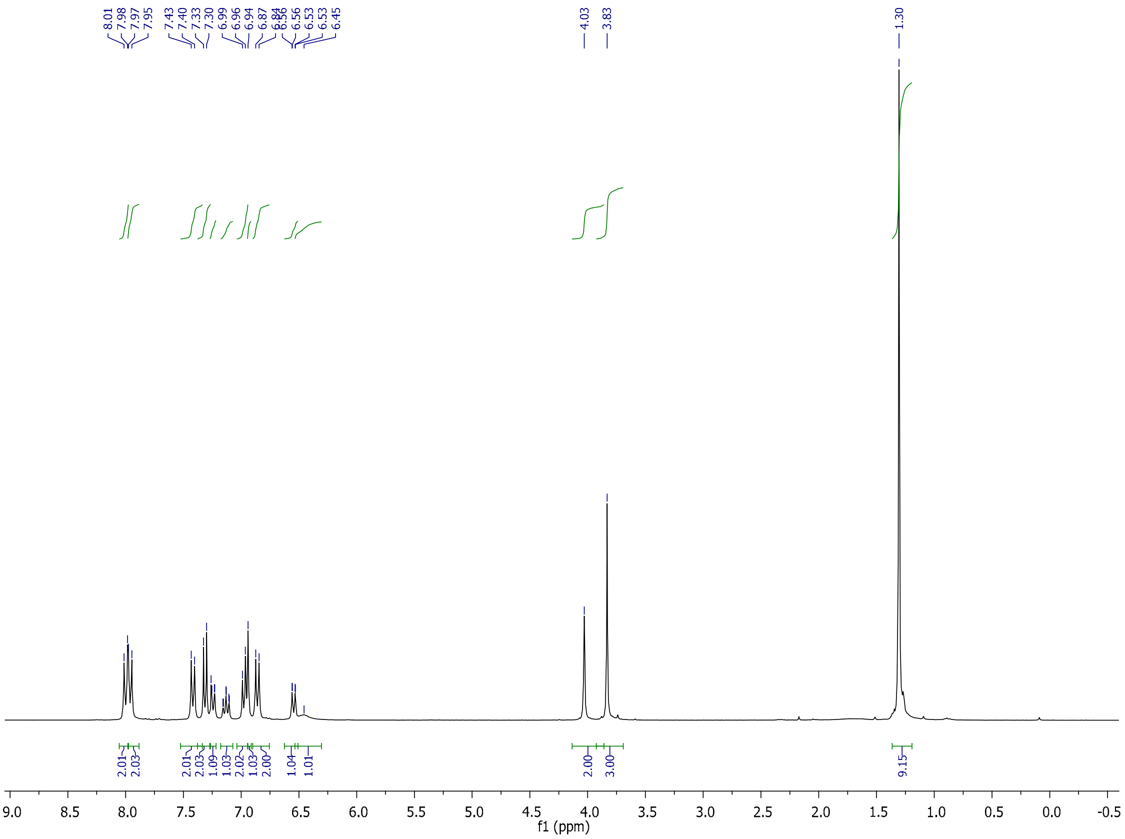
8.01
7.98
7.97
7.95
7.43
7.40
7.33
7.30
6.99
6.96
6.94
6.87
6.53
6.45
4.03
3.83
1.30
2.01
2.03
1.09
1.03
2.02
1.03
2.00
1.04
1.01
3.00
9.15
f1 (ppm)

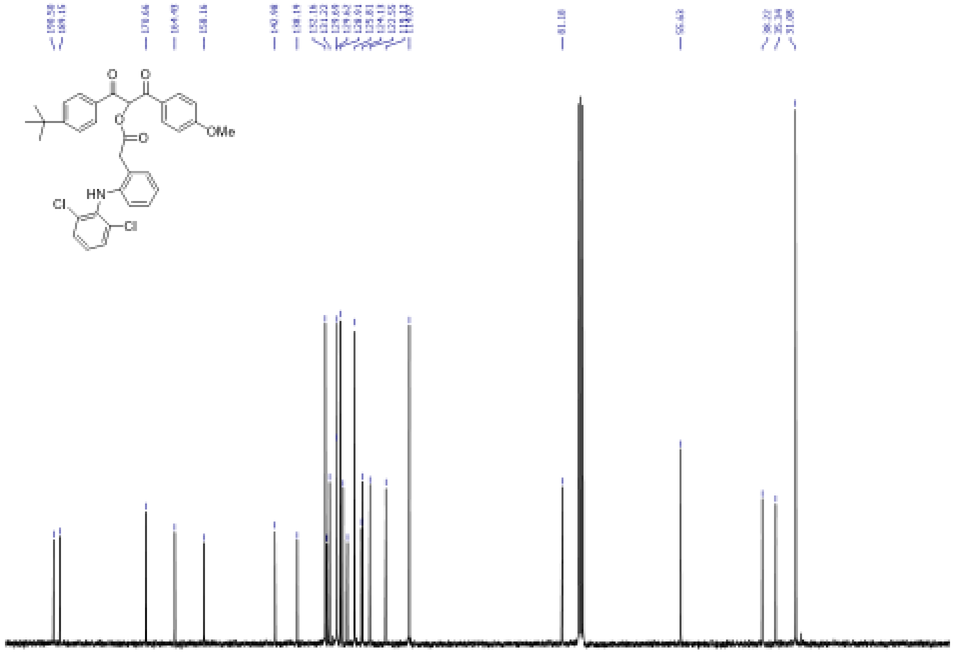
OMe
HN
Cl
Cl
f1 (ppm)

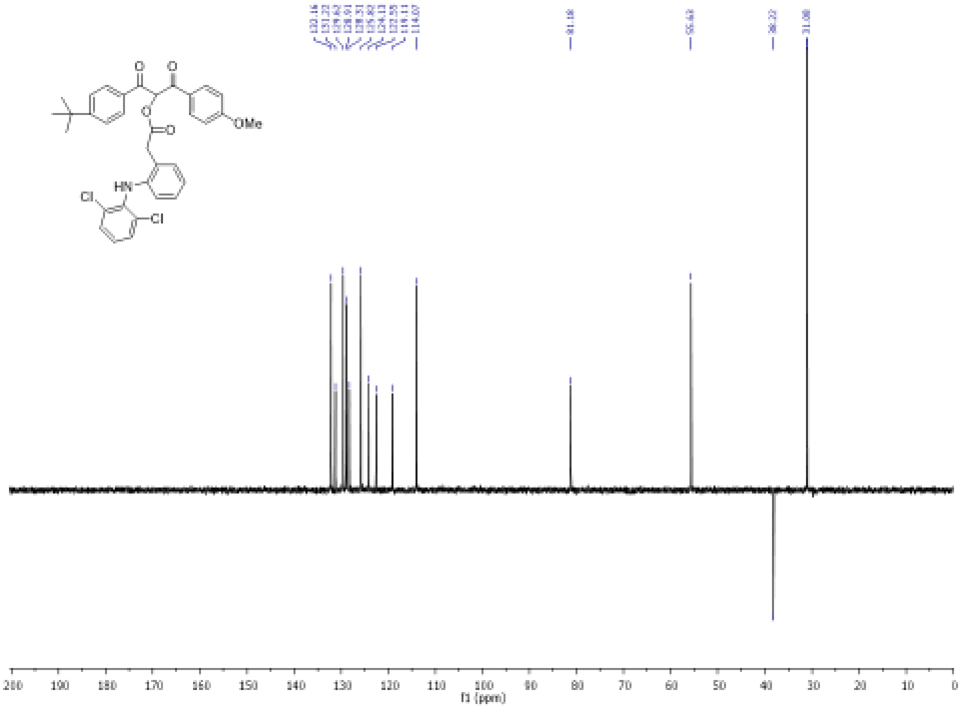

81.18
55.63
38.22
31.08
OMe
HN
Cl
Cl
f1 (ppm)

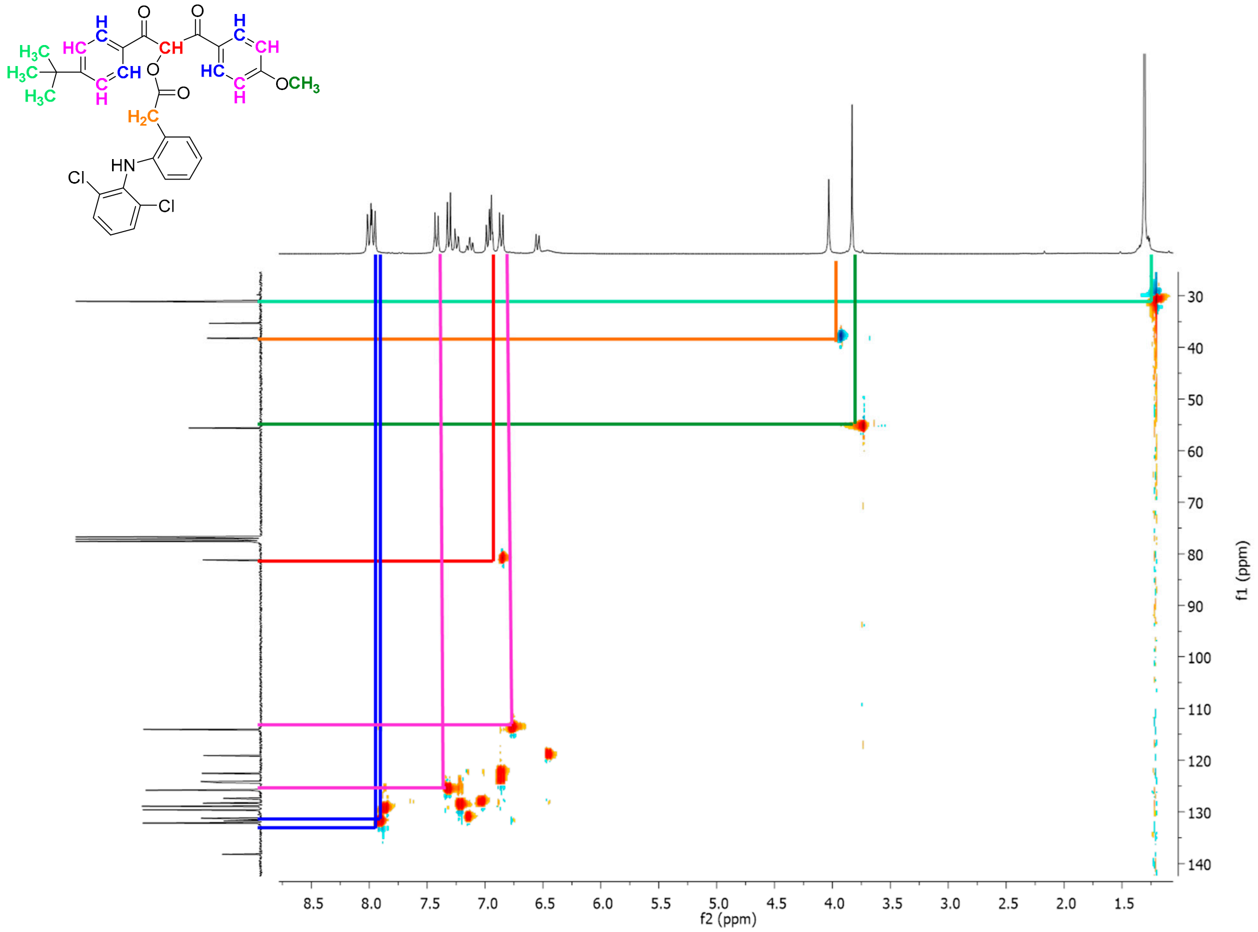

OCH3
HN
Cl
Cl
f2 (ppm)
f1 (ppm)

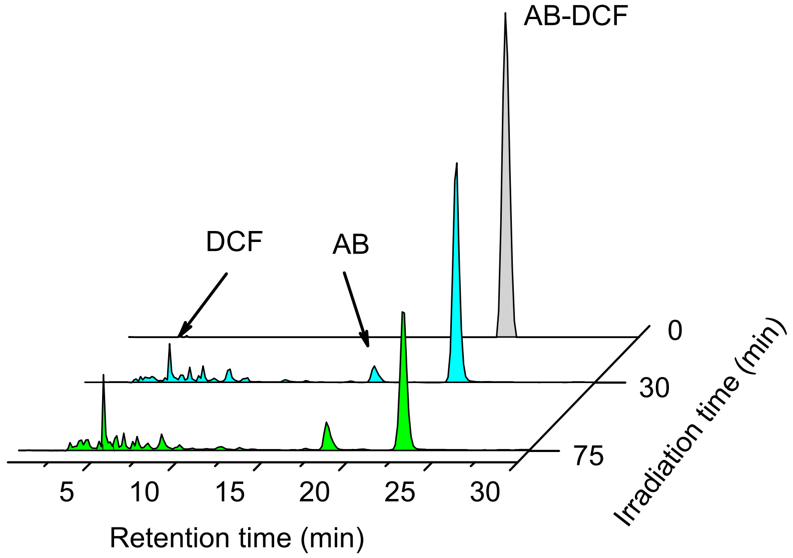

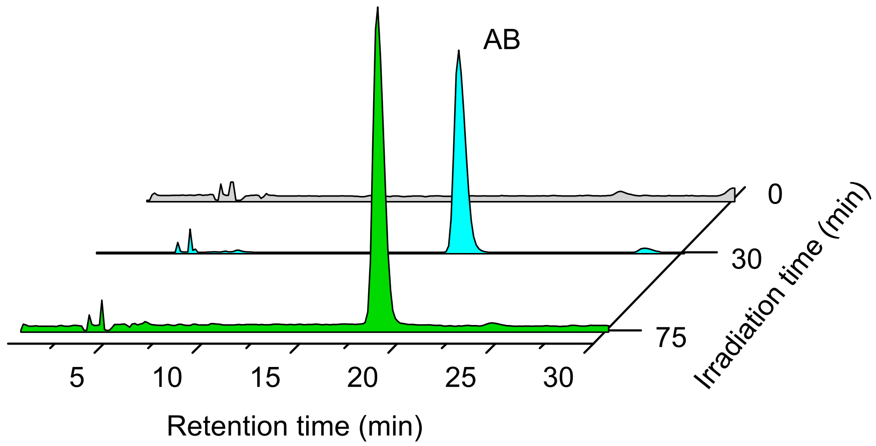

Chromatograms of an ethanol solution of AB–DCF irradiated with SSL under N2 atmosphere, detection at 274 nm (top) or 357 nm (bottom).
